# The Teach-Back Effect on Self-Efficacy in Patients with Type 2 Diabetes

**DOI:** 10.1900/RDS.2020.16.46

**Published:** 2021-05-01

**Authors:** Marhamat Farahaninia, Tahere Sarboozi Hoseinabadi, Rasool Raznahan, Shima Haghani

**Affiliations:** 1 Department of Community Health Nursing, School of Nursing and Midwifery, Iran University of Medical Sciences and Health Services, Tehran, Iran.; 2 Department of Nursing, School of Nursing and Midwifery, Torbat Heydariyeh University of Medical Sciences, Torbat Heydariyeh, Iran.; 3 Health Sciences Research Center, Torbat Heydariyeh University of Medical Sciences, Torbat Heydariyeh, Iran.; 4 Biostatistics Nursing Care Research Center, Iran University of Medical Sciences, Tehran, Iran.

**Keywords:** teach-back, self-efficacy, self-management, training, type 2 diabetes, disease management

## Abstract

**BACKGROUND:**

Diabetes is a chronic, metabolic disease, which is commonly associated with increased blood glucose levels caused by impaired secretion or function of insulin. Therefore, daily blood glucose control, adherence to a dietary and pharmaceutical regimen, regular physical activity, and foot care are fundamental components of disease management. In order to optimize effective self-management, patients need to be trained. Teach-back is a method which aims to improve patients’ understanding and perception of treatment regimens based on the interaction between patient and caregiver.

**AIM:**

This study was conducted to investigate the impact of the teach-back method on the effectiveness of self-management in patients with type 2 diabetes (T2D).

**METHODS:**

A total of 74 patients with T2D were included in the study by convenience sampling at the Endocrine and Metabolism Clinic. The subjects were assigned to control or intervention group. Data collection was performed by using a demographic data form and a self-efficacy questionnaire that were provided to the patients before and 1 month after training. The patients in the intervention group received a 5-session training program using the teach-back method. The control group received only routine programs. One month after completion of the training sessions, the questionnaires were completed by the subjects in the 2 groups, and the data obtained were analyzed.

**RESULTS:**

In contrast to the control group, mean and standard deviation of self-efficacy were significantly higher in the intervention group one month after training by the teach-back method than before training. The two groups did not significantly differ regarding mean score of self-efficacy before training, but there was a significant difference one month after training: the mean score of self-efficacy in the intervention group was significantly higher than in the control group (p < 0.001).

**CONCLUSIONS:**

Teach-back is a training procedure aimed at improving patients’ understanding of treatment regimens. This study showed that teach-back significantly improved patients’ self-efficacy even over as short a period as one month. It may be interesting to study the long-term effects of this simple but effective training method.

## 1 Introduction

Chronic diseases are among the main causes of mortality and disability in the world today [1]. Decline in physical activity, increase in obesity and tobacco use, and increasingly aging populations have led to considerable growth rates in the prevalence of chronic diseases in societies [2]. Diabetes is one of the most common chronic metabolic diseases [3, 4]. According to reports by the World Health Organization (WHO), about 422 million individuals were affected by diabetes in 2014, with greater prevalence in low and moderate income countries. The global prevalence of diabetes is estimated to be 8.5% among adults aged above 18 years. It was the seventh leading cause of mortality in 2016 (1.6 million individuals died from consequences of diabetes) [5].

Diabetes may cause short-term complications, including as hypoglycemia and diabetic ketoacidosis, and long-term complications, including retinopathy, nephropathy, neuropathy, and cardiovascular disease [6]. It may also lead to disability and a multi-year decline in life expectancy [7, 8]. Therefore, daily blood glucose control, adherence to a dietary and pharmaceutical regimen, physical activity, and regular foot care are essential factors in disease management [9, 10]. About 95% of diabetes treatment consists of self-management criteria [11, 12]. Application of effective self-management behavior is thus decisive in diabetes treatment; therefore, its effectiveness needs to be optimized by training people with diabetes, which should result in improved self-efficacy [13].

Self-efficacy means self-confidence and the individual’s ability to conduct self-management in various situations [14]. Perceived self-efficacy is an important factor for successful performance of self-management and a fundamentally required skill to perform it [15]. Bandura described self-efficacy as an individual’s belief in his ability to achieve a specific goal [16]. The WHO describes self-efficacy as a measure of health promotion at an individual level to achieve control over one’s own life [17]. Improvement of self-efficacy is an important prerequisite for behavioral changes and may result in an increase in life-expectancy [18, 19].

Training is a proper tool to increase awareness in patients, so that active and informed cooperation for the self-management of their disease is improved [20]. Also, training should be based on patients’ needs and emphasize the key aspects of the teach-back method [21]. Teach-back is an evidence-based training method based on the interaction between patient and physician; it is helpful to assess patients’ understanding and perception of treatment regimens through interviewing patients; its aim is to increase patients’ knowledge and perception and improve their self-management. This method provides valuable information for patient and healthcare givers and helps to improve the treatment process and outcome [22]. It aims to provide effective learning and decrease memory errors and mistakes [23]. Teach-back is considered one of the most effective methods to improve training perception [24, 25]. In teach-back, the trainer teaches content in simple and understandable language without using medical terms, and after finishing the training, the clients are asked to explain the content as they perceived it. If the client did not understand the content properly, the trainer should repeat it to clients until complete understanding is reached [26]. The nurse trainer may access the needs of the patients involved by communication skills, and has the ability to design and present individualized training to meet these needs [27]. Since training is important for patients to ensure individuals’ perception, recall, and maintenance of training information, the aim of this study was to determine the effect of the teach-back method on self-efficacy in patients with T2D over a short one-month period to determine whether immediate success can be obtained by introducing this method.

## 2 Materials and methods

### 2.1 Patients

This was a pretest/posttest clinical trial with a control group. The study included 74 patients with type 2 diabetes (T2D) from the Endocrinology and Metabolism Clinic of Gonabad University of Medical Sciences, Gonabad City, Iran in 2018. The samples were selected by convenience sampling and according to the inclusion and exclusion criteria. Inclusion criteria included:

- Over 18 years
- No mental illnesses
- Literate
- Not part of the health workforce
- No history of participation in diabetes education programs in the last six months
- Possibility of contacting their family members

The exclusion criterion was: developing physical problems that prevent self-care. The patients were allocated to the 2 groups, namely training (n = 37) and control (n = 37).

After obtaining permission from the Ethics Committee of Iran University of Medical Sciences, the relevant data were collected by referring the patients to the Diabetes Clinic of Gonabad City. Details of the project, research objectives, and confidentiality of personal information were explained to the study participants, and written informed consent was obtained from all participants.

### 2.2 Study design

In order to prevent leakage of information, the two groups (control and intervention) had been selected separately and in isolation from each other. The selection of subjects was performed at the beginning of the week. Training sessions were held during the same week in the diabetes clinic for individual subjects on a face-to-face basis. The teach-back method was implemented by a person-to-person training program that was carried out during 4 sessions of 30 to 45 min in addition to the usual departmental program. Also, at the end of each training session, the education manuals on diabetes were provided to the intervention group. The control group received the routine program including training by a doctor or nurse accompanied by departmental posters. One month later, the two groups were evaluated.

### 2.3 Data gathering

Clinical and demographic characteristics were recorded and a diabetes self-efficacy questionnaire (DMSES) was used to assess the level of patients’ self-management of their disease. Clinical and demographic characteristics included gender, age, marital status, occupational status, educational level, residence location, income, insurance and supplementary insurance status, household, individuals living with the patient, height, weight, body mass index, tobacco use, fasting blood glucose level at last laboratory test, history of diabetes, type of treatment, and history of training. The DMSES questionnaire used for assessing patients’ self-efficacy consisted of 19 questions scored on the 11-degree Likert scale, ranging from “I cannot definitely” with zero score to “I can definitely” with a score of 10. The patients scored themselves from zero to ten for each question to assess their performance and awareness in the context, with higher scores representing higher self-efficacy. The scores obtained represented the following degrees of self-efficacy:

1. 0-66: low self-efficacy
2. 66-130: medium self-efficacy
3. 130-190: high self-efficacy

The questions were classified into five categories, including:

- Nutrition and diet: questions number 4, 5, 9, 11,12, 13, 14, 15, and 16 (nine questions)
- Physical activity: questions number 8 and 10 (two questions)
- Medication use: questions number 2, 18, and 19 (three questions)
- Measurement of blood sugar: questions number 1, 2, and 3 (three questions)
- Assessment of feet and referral to physician: questions number 7 and 17 (two questions)

The internal consistency coefficient (Cronbach’s alpha) was 0.83.

### 2.4 Data analysis

The collected data were analyzed by SPSS. Quantitative data were represented by mean and standard deviation. Frequency distribution tables and related diagrams (for qualitative data) were used to describe the results. The Kolmogorov-Smirnov test was used to assess normality of data. Paired sample t-test and independent t-test were applied to compare mean scores and normal data. Non-parametric Mann-Whitney U-test and Wilcoxon signed-rank test were used for analyzing non-normal data. The significance level was considered 0.05 for all tests.

## 3 Results

The data obtained suggested that the control and intervention group were homogeneous in terms of clinical and demographic information. The mean score for age was 47.08 years in the intervention group and 43.54 years in the control group, with a mean age of 45.31 years in both groups. The majority of subjects were married and homemakers, held a diploma or degree, and had a history of diabetes of longer than 2 years (Table 1).

**Table 1.** Clinical and demographic information on type 2 diabetic patients in the intervention and control group

		Intervention	Control	Test results
		n (%)		
Age (yr)	20-39	11(29.8)	15(40.6)	t = 1.4 df = 72 p* = 0.16
	40-59	19(51.3)	17(45.9)	
	60-70	7(18.9)	5(13.5)	
	Mean ± SD	47.08 ± 10.39	43.54 ± 11.20	
Gender	Male	18(48.6)	18(48.6)	x^2^ = 0 df = 1 p** = 1
	Female	19(51.4)	19(51.4)	
Marital status	Married	30(81)	23(62.1)	x^2^ = 3.2 df = 1 p** = 0.07
	Single	7(19)	14(37.9)	
Employment status	Employed	3(8.2)	4(10.8)	p*** = 0.96
	Self-employment	8(21.6)	9(24.3)	
	Retired	5(13.5)	3(8.2)	
	Homemaker	12(32.4)	11(29.7)	
	Unemployed	5(13.5)	7(18.9)	
	Worker	4(10.8)	3(8.1)	
Education	Illiterate	6(16.2)	6(16.2)	x^2^ = 4.98 df = 4 P** = 0.28
	Primary school	9(24.4)	9(24.4)	
	Secondary school	8(21.6)	7(18.9)	
	Diploma	12(32.4)	7(18.9)	
	Undergraduate or higher degree	2(5.4)	8(21.6)	
History of diabetes	6 months to 2 years	6(16.3)	8(21.7)	X^2^ = 0.45 df = 2 p** = 0.79
	2-4 years	13(35.1)	11(29.7)	
	>4 years	18(48.6)	18(48.6)	
	Mean ± SD	2.32±0.74	2.27±0.80	

Legend: * Independent t-test, ** chi-square test, *** Fischer’s exact test

According to independent t-test, mean ± SD scores for self-efficacy before training were 95.89 ± 10.77 in the intervention group and 95.54 ± 10.97 in the control group. There were no significant differences in self-efficacy before training between the 2 groups. However, the difference between the two groups was significant one month after training, with 148.51 ± 19.78 in the training group and 97.95 ± 15.72 in the control group (p < 0.001). This result was also confirmed by the paired sample t-test: the difference between scores before and after 1 month of training was significant in the intervention group, but not the control group (t = 13.94, p < 0.001) (Table 2).

**Table 2.** Numerical indicators of self-efficacy in patients with type 2 diabetes in the intervention and control group

Time	Intervention group	Control group	Independent t-test
	Mean ± SD		
Before training	95.89 ± 10.77	95.54 ± 10.97	t = 0.13, df = 72, p = 0.89
One month after training	148.51 ± 19.78	97.95 ± 15.72	t = 12.17, df = 72, p < 0.001
Paired t-test	t = 13.94, df = 36, p < 0.001	t = 0.76, df = 36, p = 0.45	

According to independent t-test, mean scores for self-efficacy before training were not significantly different between the 2 groups. However, one month after training, the difference between the groups was significant; the mean scores were significantly higher in the intervention group (p < 0.001). According to the paired t-test, there was also a significant difference between the mean scores for self-efficacy before and after 1 month of training in the intervention group; they were significantly higher 1 month after training (p < 0.001). Meanwhile, there was no significant difference in the control group (Table 3).

**Table 3.** Numerical indicators of self-efficacy in patients with type 2 diabetes in the intervention and control group

Self-efficacy	Time	Intervention group	Control group	Independent t-test
		Mean ± SD		
Nutrition and diet (0-90)	Before	41.2 ± 5.8	40.5 ± 6.2	t=0.52, df=72, p=0.60
	After	62.2 ± 9.1	40.4 ± 9.2	t=10.23, df=72, p<0.001
Paired t-test		t=12.3, df=36, p<0.001	t=0.98, df=36, p=0.01	
Physical activity (0-20)	Before	10.2 ± 2.2	10 ± 2.2	t=0.42, df=72, p=0.67
	After	16 ±3.1	9.6 ± 2.6	t=9.43, df=72, p<0.001
Paired t-test		t=11.34, df=36, p<0.001	t=0.60, df=36, p=0.55	
Medication use (0-30)	Before	14.7 ± 3	14.8 ± 2.3	t=0.08, df=72, p=0.93
	After	23.3 ± 3.4	16.5 ± 2.7	t=8.02, df=72, p<0.001
Paired t-test		t=8.98, df=36, p<0.001	t=3.32, df=36, p=0.002	
Measurement of blood glucose (0-30)	Before	15 ±3.6	14.5 ± 3.1	t=0.58, df=72, p=0.55
	After	23.1 ± 4.8	16.9 ± 4.8	t=5.52, df=72, p<0.001
Paired t-test		t=7.98, df=36, p<0.001	t=2.81, df=36, p=0.008	
Assessment of feet and referral to physician (0-20)	Before	10.1 ± 2	10.1 ± 1.3	t=0.06, df=72, p=0.94
	After	15.3 ± 3.1	10 ± 3	t=7.48, df=72, p<0.001
Paired t-test		t=8.31, df=36, p<0.001	t=0.26, df=36, p=0.79	

## 4 Discussion

The results suggested that a one-month teach-back training significantly improved self-efficacy in the intervention group, while there was no significant difference between mean and SD scores in the control group before and after training. Inter-group comparisons revealed that the intervention group performed significantly better: mean and SD of self-efficacy were significantly higher in the intervention group after the one-month training, while there was no significant difference between the groups before training. This result is in accordance with other studies showing that performing training interventions results in significant improvements in self-efficacy [28-33].

In the study by Naghibi *et al*., self-care of patients in both groups was significantly increased after intervention, but the increase was greater in the control group [34]. The authors concluded that the significant self-care improvement in the control group was due to training in service-providing centers and external factors such as media, relatives’ awareness, and patients’ reading, which increased their self-care [34]. This is consistent with our results and with those obtained in the other studies confirming that better awareness and knowledge improve self-efficacy.

Self-efficacy is a means for an individual to perform a specific behavior that is beneficial for diabetes management and for obtaining expected results. Bandura *et al*. believe that the feeling of self-efficacy is formed as result of enduring challenges and sequential, step-by-step performance of the behavior. They stated that self-efficacy is the main and most important prerequisite of behavioral change, and that it is one of the beneficial health behaviors [35]. Since diabetes is a chronic disease, treatment success requires active and continuing cooperation of the patient in the treatment process, and since daily activities, lifestyle, and dietary habits of the patient have a considerable effect on glycemic control, training of the patient is vitally important. Correct training may result in the prevention or at least reduction of long-term complications and hospitalization. Teach-back is a specific training method aimed at improving patients’ perception, and it is applicable in the context of diabetes disease management. Increased knowledge enables the patient to achieve better self-care and may lead to informed decision-making related to the continuity of self-care and eventually a decline in physical and mental complications [36].

There are limitations to this study. The educational levels of the participants were heterogeneous which may have had an impact on the answers. Also, participants sometimes had difficulties attending training classes. In these cases, it was necessary to work more closely with them to solve this problem.

## 5 Conclusions

Health education and appropriate corrective and behavioral approaches are among the most effective ways of preventing and controlling diabetes. These strategies focus on raising awareness of medical needs to achieve optimal glycemic control and on reinforcing the motivation and skills of individuals to engage in the implementation of prescribed therapies and participate actively in self-care with the help of other family members. Considering the efficacy of the intervention to improve the lifestyle of people with type 2 diabetes, rehabilitation in the field of diabetes is feasible and an effective way to improve the status of people with diabetes. Training by the teach-back method has been shown to be a feasible and effective means of implementing self-efficacy and thus improving lifestyle changes for optimized disease management.
